# A novel spherical ultrasonic motor with wire stators and measuring torque and preload via a new method

**DOI:** 10.1038/s41598-023-39111-8

**Published:** 2023-07-24

**Authors:** Seyed Hassan Jahantab, Yousef Hojjat, Behzad Ghavami Namin, Mohammad Shirkosh

**Affiliations:** Department of Mechanical Engineering, Tarbiat Modares University, Tehran, Iran

**Keywords:** Mechanical engineering, Electrical and electronic engineering

## Abstract

The present study introduces a multi-degree-of-freedom (MDOF) ultrasonic motor, which is capable of driving a spherical rotor using spiral wire stators and a piezoelectric stack actuator. Wire stators and piezoelectric stack actuators enable the proposed motor to be smaller and simpler, lower in power consumption, and have different modes at different frequencies. In this motor, two wire stators are used to drive the spherical rotor and rotate it in different directions. The eigenfrequency and frequency domain analyses were carried out using the finite element method (FEM) to evaluate the MDOF capability of the motor in different vibration modes. It has been demonstrated that the piezoelectric stack actuator can provide MDOF motions through its vibration modes. The resonant frequency obtained by the frequency domain approach agreed with the impedance analyzer test. Rotational speed, torque, and preload force were experimentally investigated. Using shear stress caused by viscous fluid in contact with the spherical rotor, a new torque calculation method was developed. Based on the buoyancy force exerted on the immersed rotor, the preload force was measured. The experimental results indicated that the maximum rotational speed of the spherical rotor was 306 rpm, and the maximum torque was 4.7 μN m.

## Introduction

Ultrasonic motors are one of the main applications of the inverse piezoelectric effect. They turn the undulatory vibrational and progressive motions of the stator (the friction between rotor and stator) into a rotary or linear motion. In recent years, high-tech applications require small, accurate, light, and low-noise actuators capable of functioning in electromagnetic environments. Ultrasonic motors are developed to address such needs. Spherical ultrasonic motors (SUSMs) have been researched and developed to benefit from the advantages of ultrasonic motors while no complex design change is required^1–7^.

Some advantages of ultrasonic motors are as follows: (1) high positioning accuracy, (2) short responding time, (3) noiseless performance, (4) high energy density, (5) simple structure, (6) high efficiency in strong magnetic fields, and (7) low power consumption^8–13^. The advantages have made ultrasonic motors a proper choice for many applications such as precise positioning, robot joints^14^, the inspection of limited-access pipes^15^, and medical applications for tiny mechanisms^16^, especially in diagnostic methods such as angioscopy, in which a spherical ultrasonic micro-motor is used to move the camera in any direction for physicians to see inside the blood vessels^17–19^. Various designs have been proposed for SUSMs, each of which has specific advantages and disadvantages regarding their potential applications and expected performance. One of such designs is SUSMs with their stator being a sandwich transducer operating in a bending mode^20–24^. Providing the proper actuation of piezoelectric material, the bending of various planes leads to rotation around different axes; however, there has been no report on simultaneous rotations around different axes. Toothed stators are a highly flexible design enabling the spherical rotor to rotate around different axes; however, it is challenging for such designs to apply the preload equally^8,25,26^. Multi-degree-of-freedom (MDOF) SUSM with four toothed piezoelectric plates, allowing for equal exertion of preload force on the rotor, was examined in another approach and correlation between rotor rotation rate and preload was also investigated^27^. Each toothed stator requires a set of at least three individual phases for excitation voltage, which may complicate the driving circuit for a higher degrees of freedom. Another SUSM design, called sandwich SUSMs, uses circular piezoelectric plates divided into several zones. Each zone must be separately excited to rotate the spherical rotor. In aforementioned study, a piezoelectric disk was adopted, and loading (the force between the rotor and stator), different contact surfaces, optimal voltage and frequency, and the friction between the spherical rotor and stator were assessed. Note that the motor structure is complicated and large regarding its efficiency^2^. Another type of SUSM was proposed, utilizing bulk piezoelectrics to serve as its actuation system. They have copper electrodes on four sides for excitation and use a combination of two perpendicular flexural vibrations to generate a motion wave on the top of the stator, leading to movement in the direction perpendicular to the bending planes^28^. In this motor the preload is applied to a soft magnetic rotor using external magnetic field, therefore application of this motor in environments with electromagnetic fields is not feasible. SUSMs with wire stators are another type using single or multi-spiral wire stators with six transducers for excitation. Because of the few contact points on the rotor, SUSMs with single spiral wire stators generate low torque; however, it is quite the opposite for SUSMs with multi-spiral wire stators. This is while the latter suffers from deviation of rotation direction due to the nonuniformity of contact points^17,18^.

Torque measurement in SUSMs is also investigated in several researches. Conventional methods like applying the torque using weight force due to hanging mass is challenging because of the geometry of the spherical rotor. Moreover, these motors provide low power and it is difficult to prevent the deviation of the rotation axis while applying the torque^18,29,30^. Indirect methods like torque estimation by measuring the transient response of the motor are also proposed^31^. The drawback of this method is the simple linear model for the relation between torque and rotational speed (load characteristic) alongside its sophisticated measurement method, which could be inaccurate for different SUSM designs.

In this study, the size and design complexity of the motor were reduced by separating the vibrating actuator from the rotor using wire stators and a piezoelectric stack actuator for the first time, resulting in two main advantages: first, high rotational speed and second, low power consumption. Novel methods were proposed to measure the torque and preload force in the present study to overcome the limitations of conventional ones. The torque was calculated using the viscous shear stress exerted on the immersed spherical rotor, and the preload force was calculated using the buoyancy force applied to the buoyant rotor. The piezoelectric stack vibrations and their different modes were numerically analyzed to determine the piezoelectric stack capabilities and the frequency response of the piezoelectric stack and stator.

## Motor structure and the governing principles

**Figure 1:**
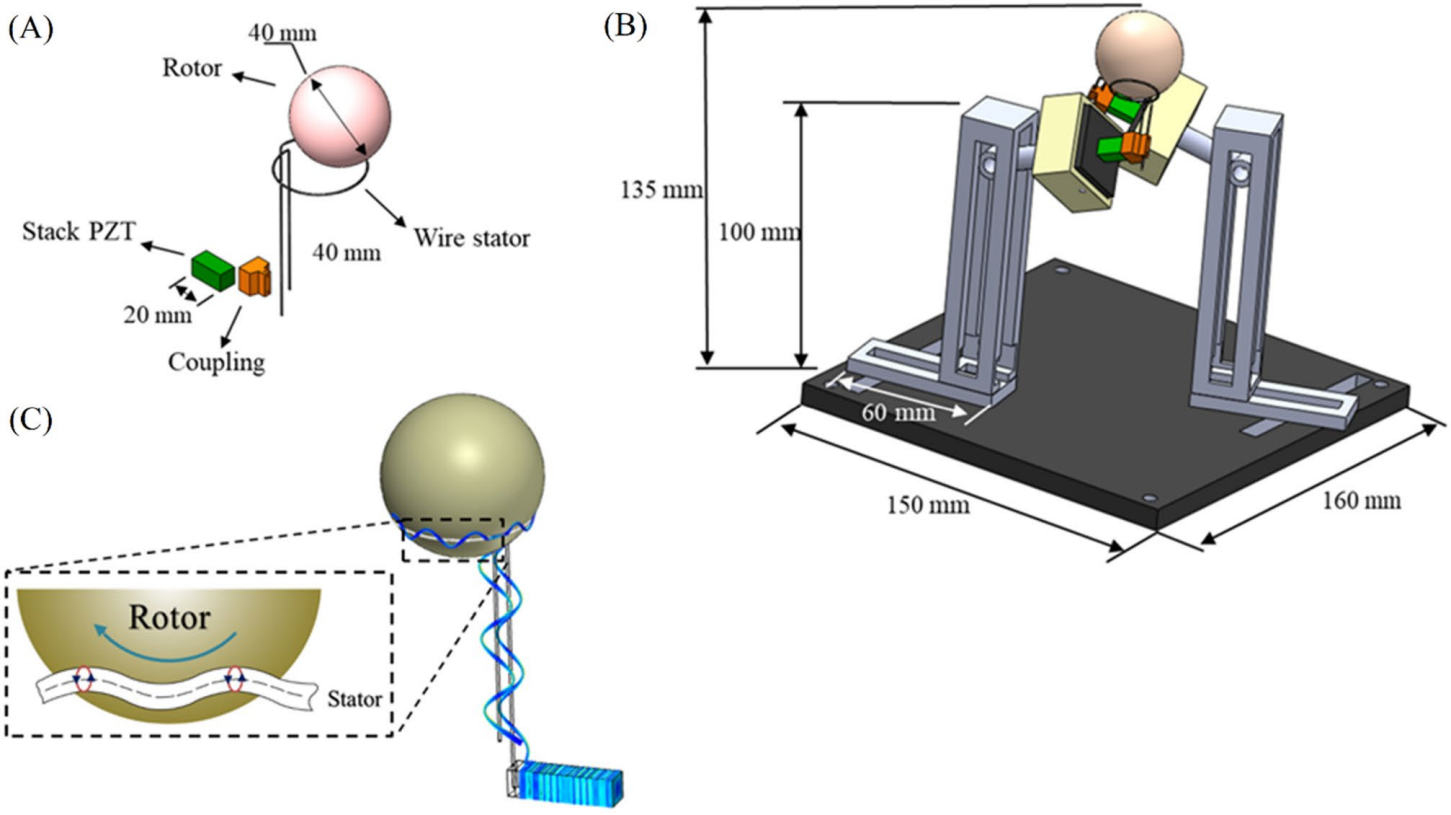
(**A**) Exploded, (**B**) assembled view of the proposed motor and (**C**) schematic of the working principle.

The motor structure and schematic of the working principle were presented in Fig. 1. The actuator is an integrated piezoelectric stack (or multilayer piezoelectrics) without sliding or rolling components. Piezoelectric stacks or multilayer piezoelectrics have a quick response (100 times faster than bimorph piezoelectrics), provide accurate positioning in nanometers, produce large forces with low energy consumption, generate no noise in electromagnetic fields, and are relatively inexpensive. Accordingly, they decrease the motor size and power consumption^31,32^. The piezoelectric material used in this study was TOKIN AE0505D16- an AE series resin-coated actuator of 5 × 5 × 20 mm. The wire stator was SUS304 with high elasticity and rigidity, and its smooth surface decreases the friction between the stator and the spherical rotor. The wire stator properties are mentioned in Table 1. The next step was to prepare a coupling to connect the wire stator to the piezoelectric stack actuator. The best coupling is to connect the wire stator directly to the piezoelectric stack actuator to transfer the vibration explicitly. This coupling is only possible with strong adhesives. Since it was aimed to utilize various stators for our experiments, such a coupling was not an option. Accordingly, a coupling was designed to transfer the vibration effectively and allow for readily stator adjustment. The connector needs to be highly rigid to transfer the vibration generated by the actuator to the stator completely; hence, polylactic acid (PLA) was used for the 3D printing of the coupling. A plastic ball (Ping pong ball FOX Inc.) was considered as the spherical rotor and its properties are mentioned in Table 2, and a 6 DOF fixture was employed to position the stators and adjust their heights accurately. By applying the proper voltage and frequency, a sinusoidal vibration was generated by the piezoelectric stack actuator, and a traveling transverse wave was transmitted to the wire stator by coupling, which induced an elliptical motion in the wire stator because of the wave propagation. Due to the friction between the rotor and stator, the crests of this traveling wave in the ring section of the stator pushes the rotor to move contrary to the wave propagation direction at the resonant frequency of the piezoelectric stack actuator and wire stator assembly (Fig. 1C). Since the piezoelectric stack actuator has different vibration modes (longitudinal, flexural, and torsional) at different frequencies, different waveforms could be generated in different directions for different modes.

**Table 1:** Specification of each wire stator**.**

Number of spirals	1
Outside diameter of spiral (mm)	30
Length of wave guide (mm)	40
Elastic modulus (GPa)	190
Speed of sound (m/s)	3141
Wire diameter (mm)	0.5
Material	SUS304

**Table 2:** Specification of spherical rotor**.**

Diameter (mm)	40
Weight (gr)	2.7
Material	Celluloid

## Simulation

**Figure 2:**
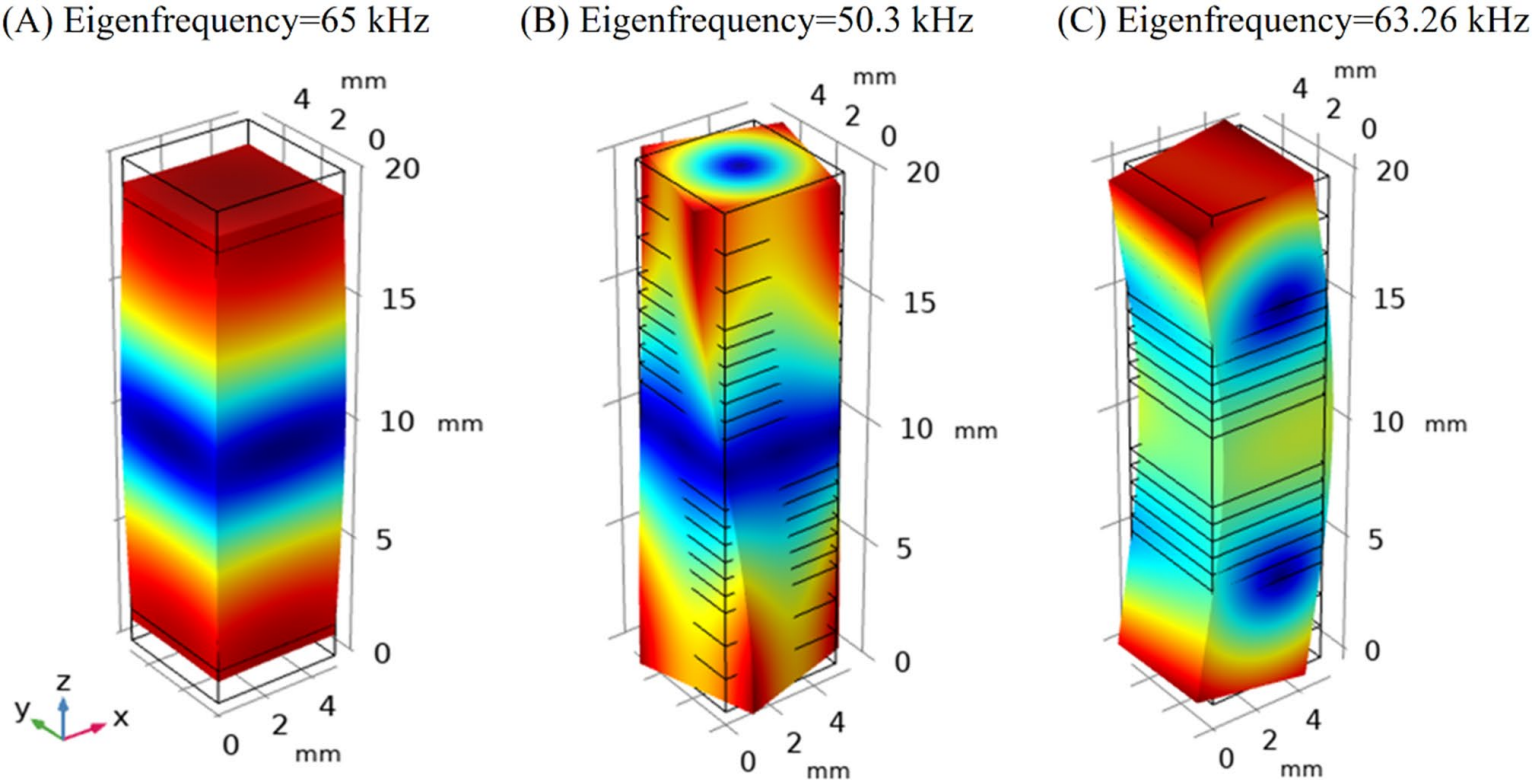
Displacement of various modes of the piezoelectric stack actuator; (**A**) longitudinal, (**B**) torsional, and (**C**) flexural.

**Figure 3:**
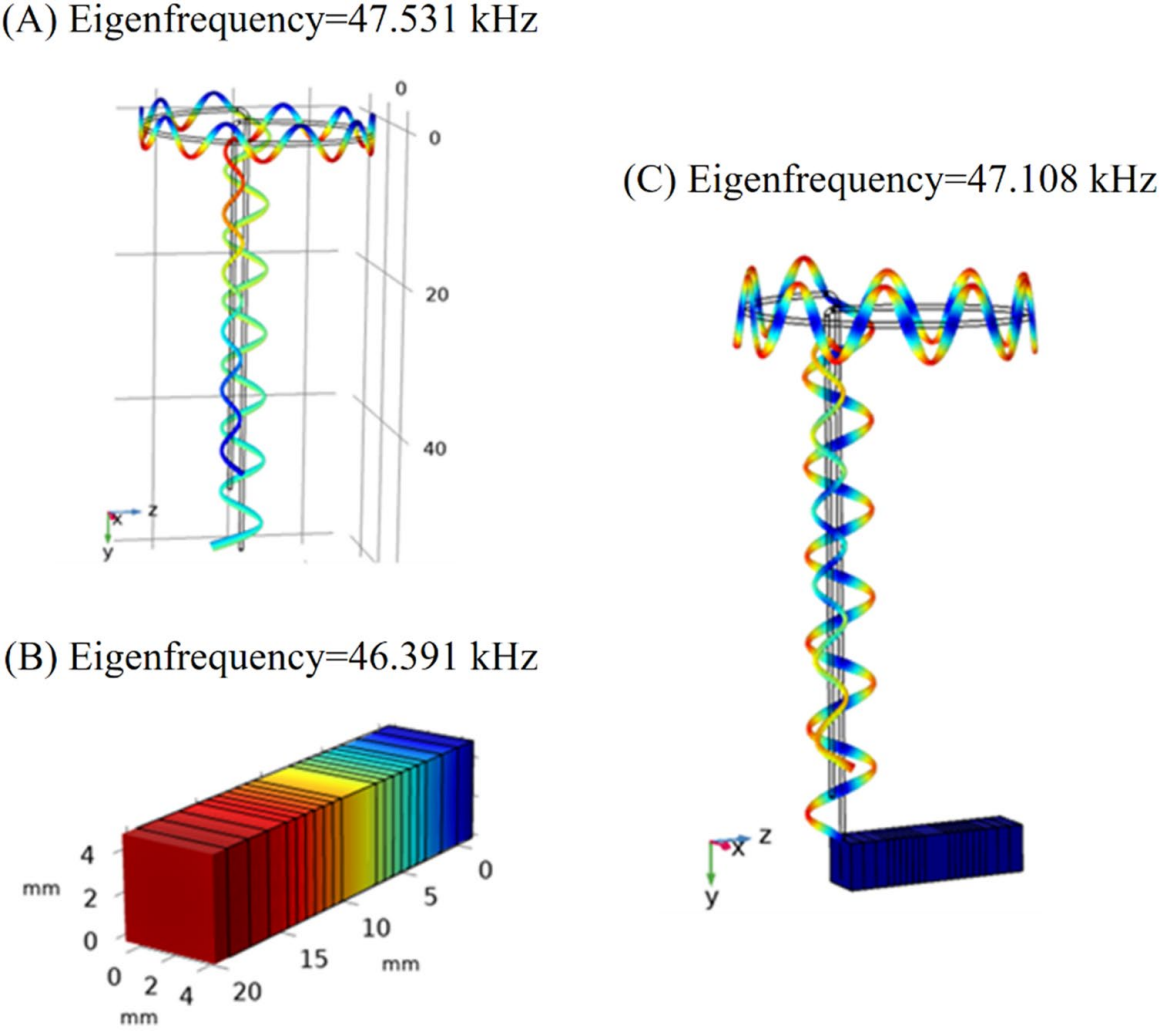
Longitudinal modes of vibration for (**A**) wire stator, (**B**) piezoelectric stack actuator, and (**C**) stator assembly.

**Figure 4:**
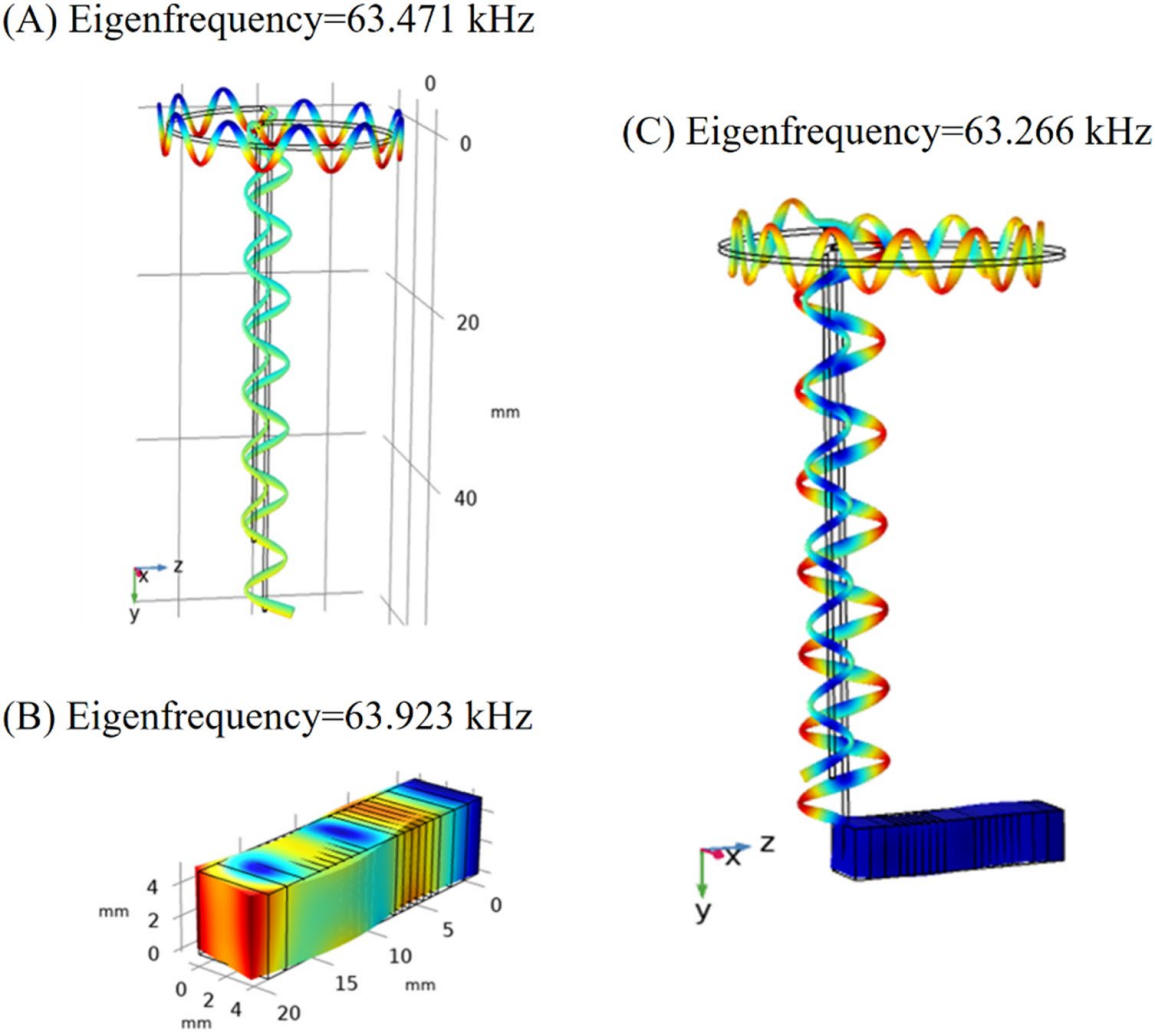
Flexural modes of vibration for (**A**) wire stator, (**B**) piezoelectric stack actuator, and (**C**) stator assembly.

**Figure 5:**
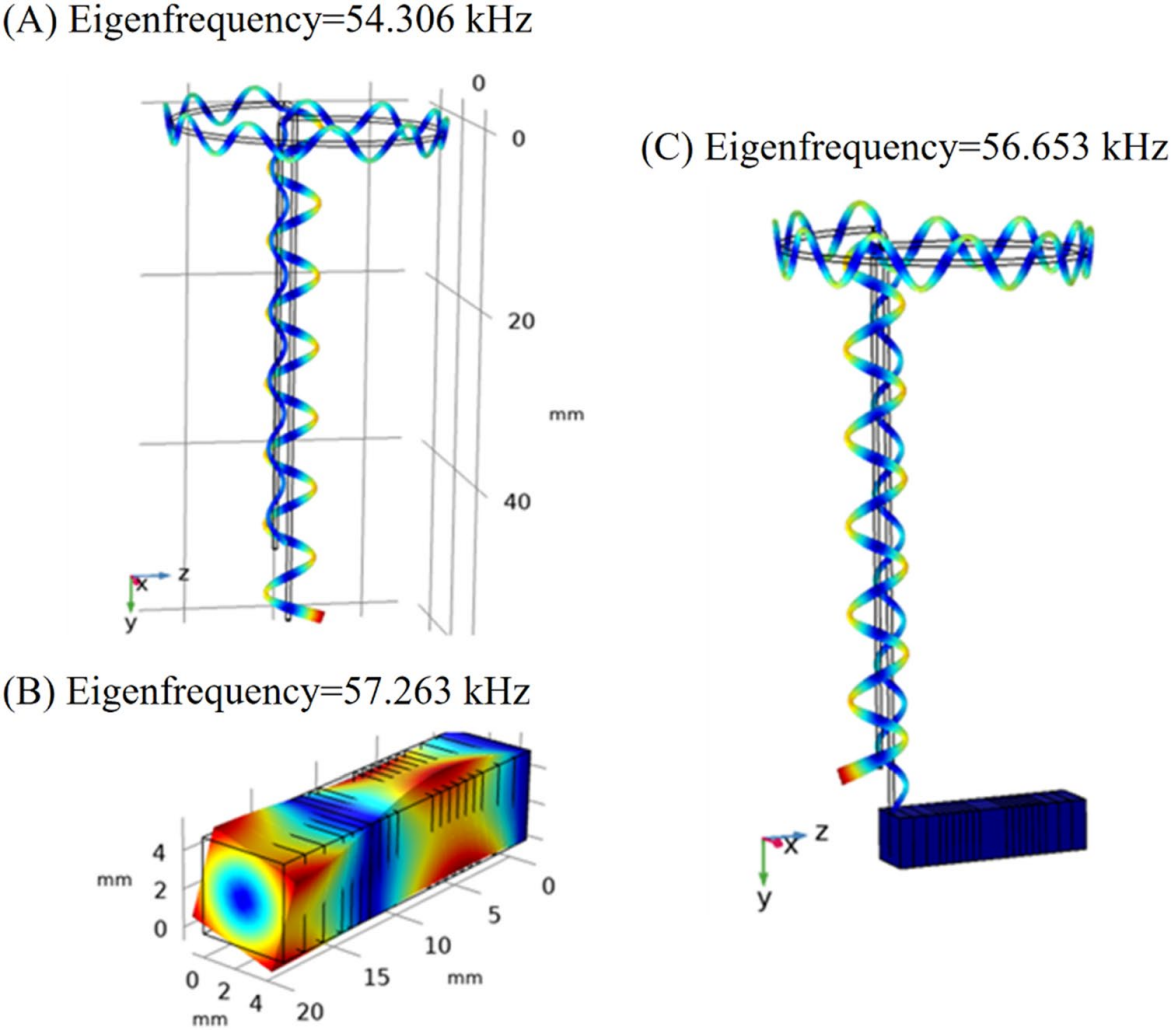
Torsional modes of vibration for (**A**) wire stator, (**B**) piezoelectric stack actuator, and (**C**) stator assembly.

A FEM simulation was conducted to evaluate the piezoelectric stack capability of making the necessary motions to actuate the stator and assess the frequency domains of vibration modes of the piezoelectric stack actuator and stator-and-wire assembly. The modal analysis was also conducted for the piezoelectric stack actuator and wire stator as a whole to determine the effect of individual vibration modes on stator motions. The piezoelectric effect is an interaction of mechanical and electrical phenomena. In COMSOL Multiphysics 6, the piezoelectric modeling includes a solid mechanic and electrostatic environments coupled by a multiphysics feature. First, the modal analysis was performed, through which the piezoelectric stack actuator was modeled as a solid in the software, according to the previous study. For the modal analysis model, the piezoelectric stack actuator was considered to be fixed at one end. The modal analysis addressed different modes of the piezoelectric stack actuator, wire stator, and stator assembly. Figures 2, 3, 4 and 5 show the results.

**Figure 6:**
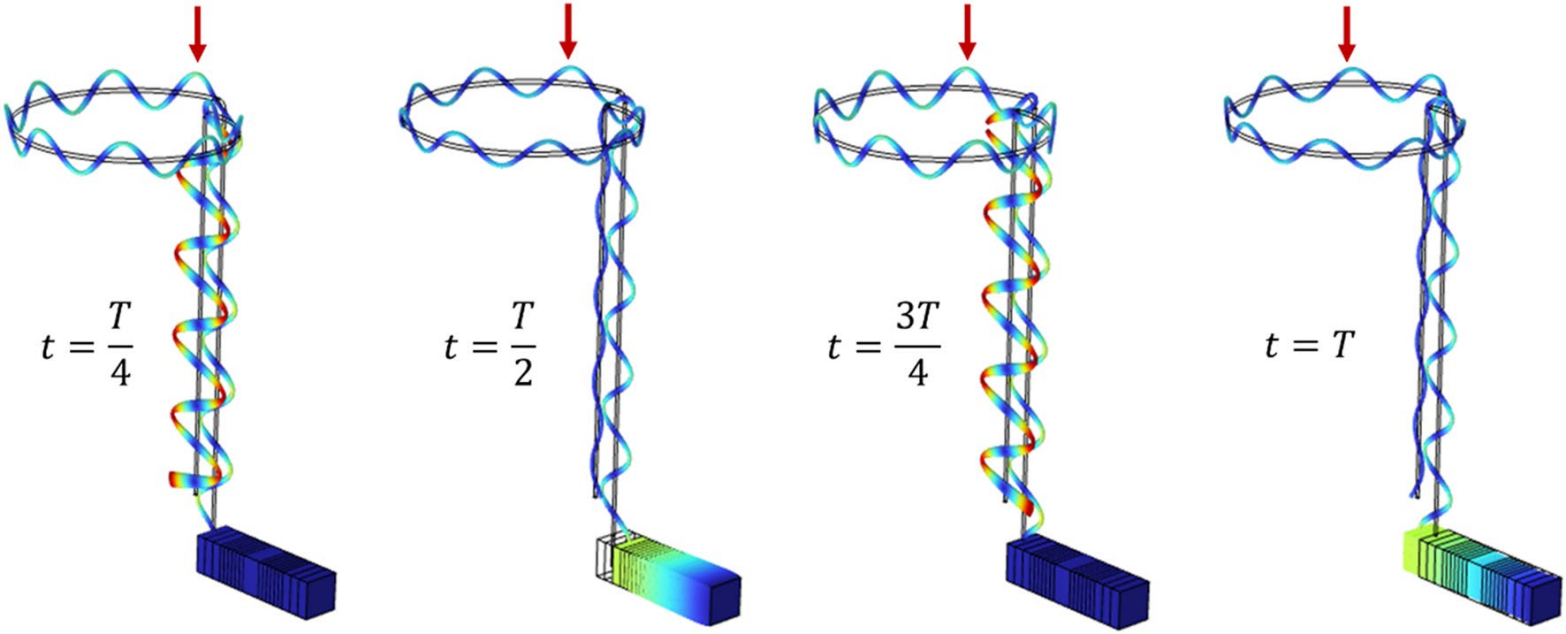
Transient motion track of the driving element.

Next, the frequency domain analysis in the sweep range of 20–80 kHz was performed for the piezoelectric stack actuator. This analysis aimed to determine the resonant frequency of the assembly when the piezoelectric stacks were actuated in the longitudinal direction. It was assumed that the electric field was applied along the 33 axis of piezoelectrics; hence, no other vibration mode was stimulated while actuating the piezoelectrics. First, harmonic analysis was carried out for the piezoelectric stacks. Then the results were regarded as the basis for the harmonic analysis of the assembly, assuming that the piezoelectric stack actuator was fixed at one end and the structural damping effects of piezoelectrics and wire stator and the dielectric and coupling of the piezoelectrics were neglectable. The amplitude of the voltage applied to the piezoelectrics was 4 V. To verify that the generated wave in the driving element is a traveling wave, a transient simulation was carried out and the results for a period of vibration were depicted in Fig. 6.

## Experimental tests

**Figure 7:**
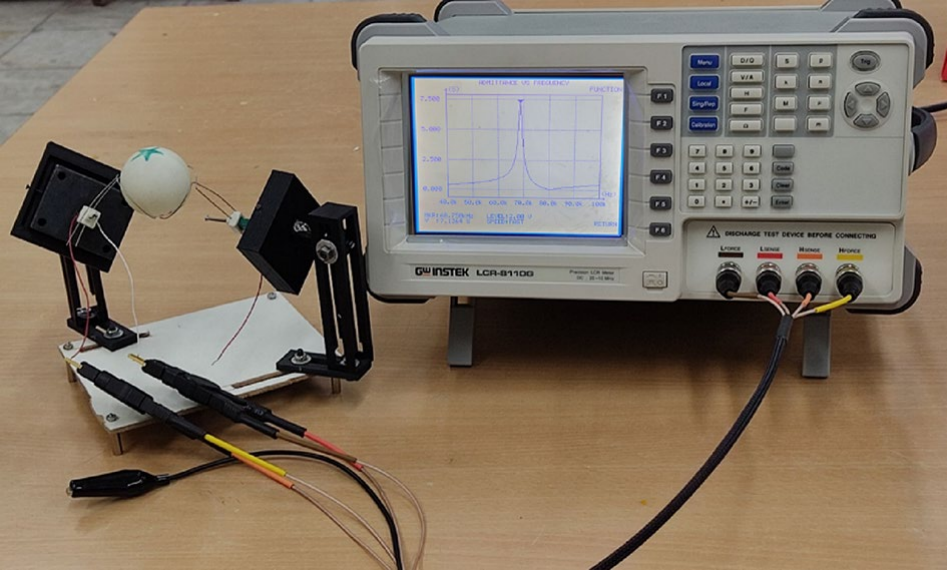
Impedance analyzer experimental test setup.

As shown in Fig. 7, the piezoelectric stack and the assembly of the piezoelectric stack, wire stators and the spherical rotor was tested by the impedance analyzer LCR-8110G to obtain the resonant frequency of the piezoelectric stack and the motor as a whole. The maximum displacement, excitation frequency, capacitance, and stiffness reported by manufacturer, were 17.5 μm, 69 kHz, 1.4 μF, and 49 N/μm under free condition, respectively.

**Figure 8:**
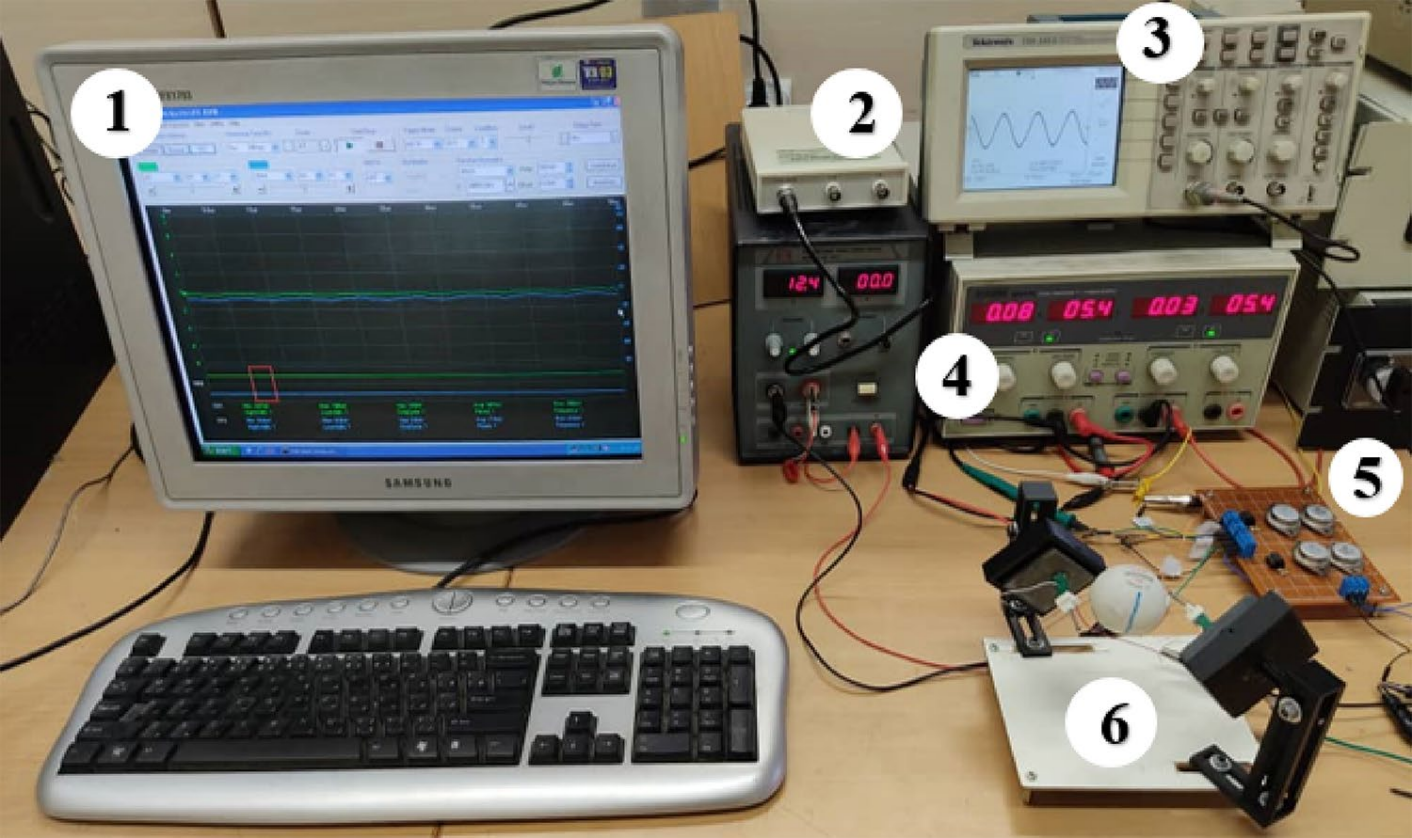
Experiment devices: (1) PC, (2) function generator card, (3) oscilloscope, (4) power supply, (5) electric circuit, and (6) rotor assembly.

The experimental setup for rotational speed measurement, including the devices used in the experiments are shown in the Fig. 8. In this study, the function generator card (TNM-DS20080A) acts as an interface between the PC and the circuit. The amplifier circuit output was connected to a class B amplifier circuit. A NE5532 integrated circuit (IC), capable of amplifying the voltage amplitude in high frequencies, was employed in the none-invert amplifier circuit. The voltage amplifier gain was 11. Piezoelectric stacks have high capacitance, and their impedance decreases in high frequencies. Accordingly, they require more current that common piezoelectric drives cannot provide. To this end, class B circuits were used to offer the additional current.

**Figure 9:**
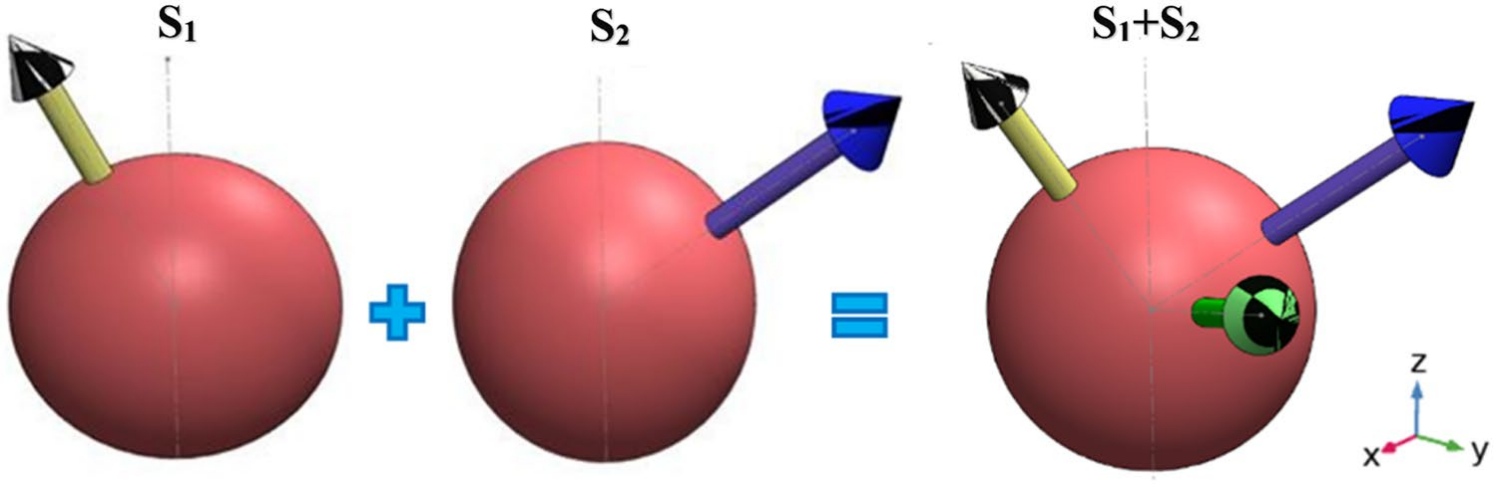
Schematic diagram of the multidirectional motion.

The impedance analyzer testing of the piezoelectric stack under free condition and its frequency domain analysis were swept in the range of 25–85 kHz. The impedance analyzer test and frequency analysis were carried out for the whole motor to derive the resonant frequency by which the motor could be started. The schematic diagram of motions and their axes for single and combination states is presented in Fig. 9. It should be noted that the green arrow in Fig. 9 depicts the axis of the resultant rotation when both stators are activated.

### Torque

**Figure 10:**
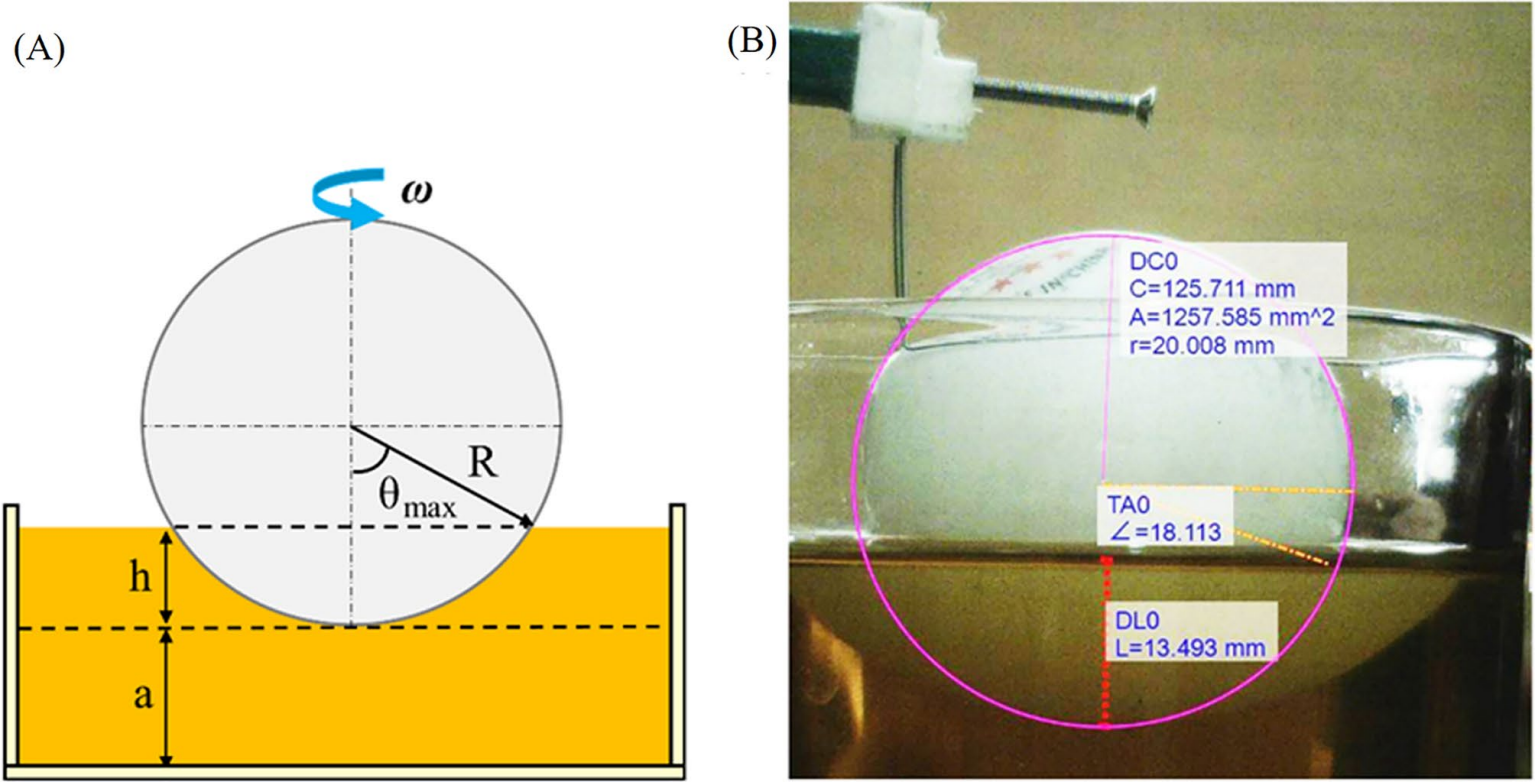
Torque measurement: (**A**) schematic and (**B**) experimental setup.

Torque is an important parameter in the performance of a motor. Due to their special geometry, the conventional torque measuring methods cannot be used for spherical rotors as such a novel approach was proposed to measure the motor's torque, addressing the issues of conventional methods. The proposed approach employed the shear stress exerted on the spherical rotor by the fluid. For this case, the stator was on top of the rotor, and a spherical cap volume of the rotor was immersed into the fluid. A highly viscous fluid (SAE 5W30 engine oil) was used to improve the accuracy of calculations. The viscosity of the oil was measured at different speeds using a rheometer. Since the rotor had low rotational velocity, *μ* = 0.26 Pa.s was selected from the rheometry diagram used for low-speed rotations. The spherical rotor speed was 12 rpm. As Fig. 10A and Eq. (2) derived in^33^ suggests that torque *T* can be calculated as follows:

1 $${\theta }_{max}={\mathrm{cos}}^{-1}\frac{R-h}{R}$$

2 $$T = \int_{0}^{\theta \max } {\frac{{\mu \omega R^{4} \sin^{2} \theta \cos \theta }}{a + R(1 - \cos \theta )}} d\theta$$

where *θ* is the spherical cap angle of the rotor, *a* is the fluid height to the lowest point of the spherical rotor, and *ω* is the rotational speed of the spherical rotor inside the viscous fluid. According to the schematic, an experimental setup was utilized to measure the required parameters in Eqs. 1 and 2 using a digital microscope (Dino-lite AM-413ZT). The desired parameters were obtained as shown in Fig. 10B.

### Preload

**Figure 11:**
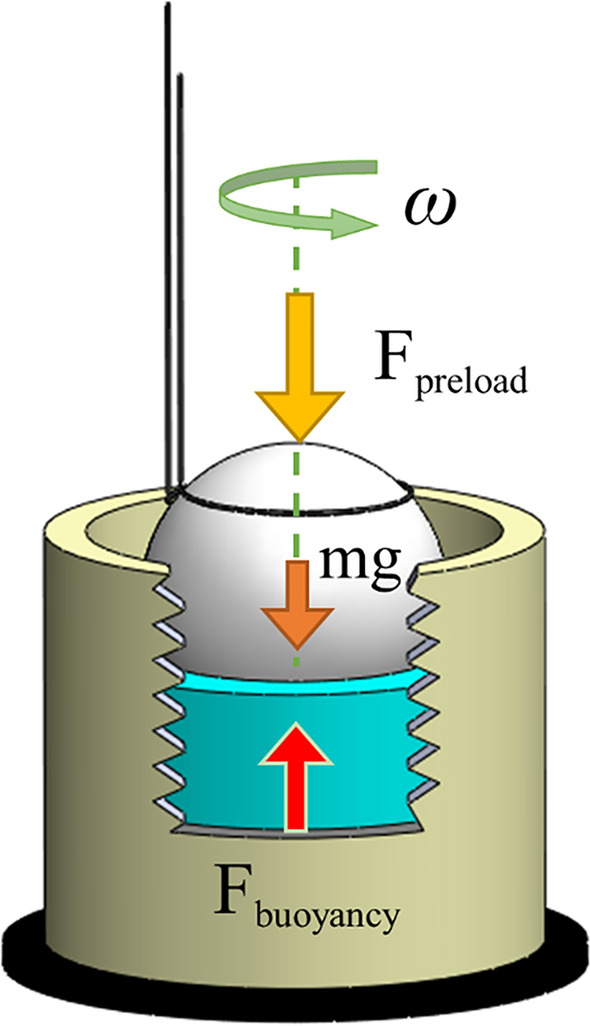
Preload measurement.

Preload measurement and the achievement of a uniform preload force for spherical rotors have always been challenging. This study used an experimental method and the buoyancy principle to measure the preload force. First, the spherical rotor was freely inside the fluid (water) without connecting to the stator. The fluid volume increased gradually, and thus, a slight contact was established between the rotor and stator. As a result, the rotor started to rotate, and as the fluid volume increased, the rotor rotated faster (due to the increased surface of contact between rotor and stator). Once the fluid reached a certain level, the rotor ceased to rotate as the excess preload, and the stator could not move the rotor (Fig. 11).

It should be noted that the friction caused by the fluid viscosity affected the rotation speed changes. As the fluid height and preload increased, the contact surface between the rotor and fluid expanded, leading to a higher viscose shear stress exerted on the rotor. In this case, the friction force between the rotor and stator and the torque caused by viscous shear stress prevented the motion of the spherical rotor. In Eq. (3), *h* is the height of the rotor inside the fluid (spherical cap), *R* is the radius of the spherical rotor, and *ρ* is the fluid density.

3 $${V}_{cap}=\frac{\pi {h}^{2}}{3}\left(3R-h\right)$$

4 $${F}_{bouyancy}=\rho g{v}_{cap}$$

5 $${F}_{preload}={F}_{bouyancy}-{F}_{gravity}$$

## Results and discussion

Figures 2A and 3B show the longitudinal modes of vibration (along the z-axis) for the free and cantilever piezoelectric stacks at the frequencies of 65 and 46.391 kHz, respectively. The torsional modes of vibration for free and the cantilever piezoelectric stack at 50.3 and 57.263 kHz are presented in Figs. 2B and 5B, respectively. Moreover, Figs. 2C and 4B illustrate the flexural vibration modes for free and the cantilever piezoelectric stack at 63.26 kHz and 63.923 in the x–z plane, respectively.

As presented in Figs. 3, 4 and 5, the frequency modes for each component and also the whole setup were evaluated and compared. Figure 3 shows the longitudinal mode for the piezoelectric stack at 46.391 kHz, the wire stator at 47.531 kHz, and the whole setup (piezoelectric stack and wire stator) at 47.108 kHz. Figure 4 shows the frequency modes for the piezoelectric stack at 63.923 kHz, the wire stator at 63.471 kHz, and the whole setup at 63.266 kHz. Figure 5 shows the torsional vibration mode for the piezoelectric stack at 57.263 kHz, frequency modes of the wire stator at 54.306 kHz, and whole setup at 56.653 kHz. All modes can provide the necessary mode shapes for the MDOF motion of the stator.

**Figure 12:**
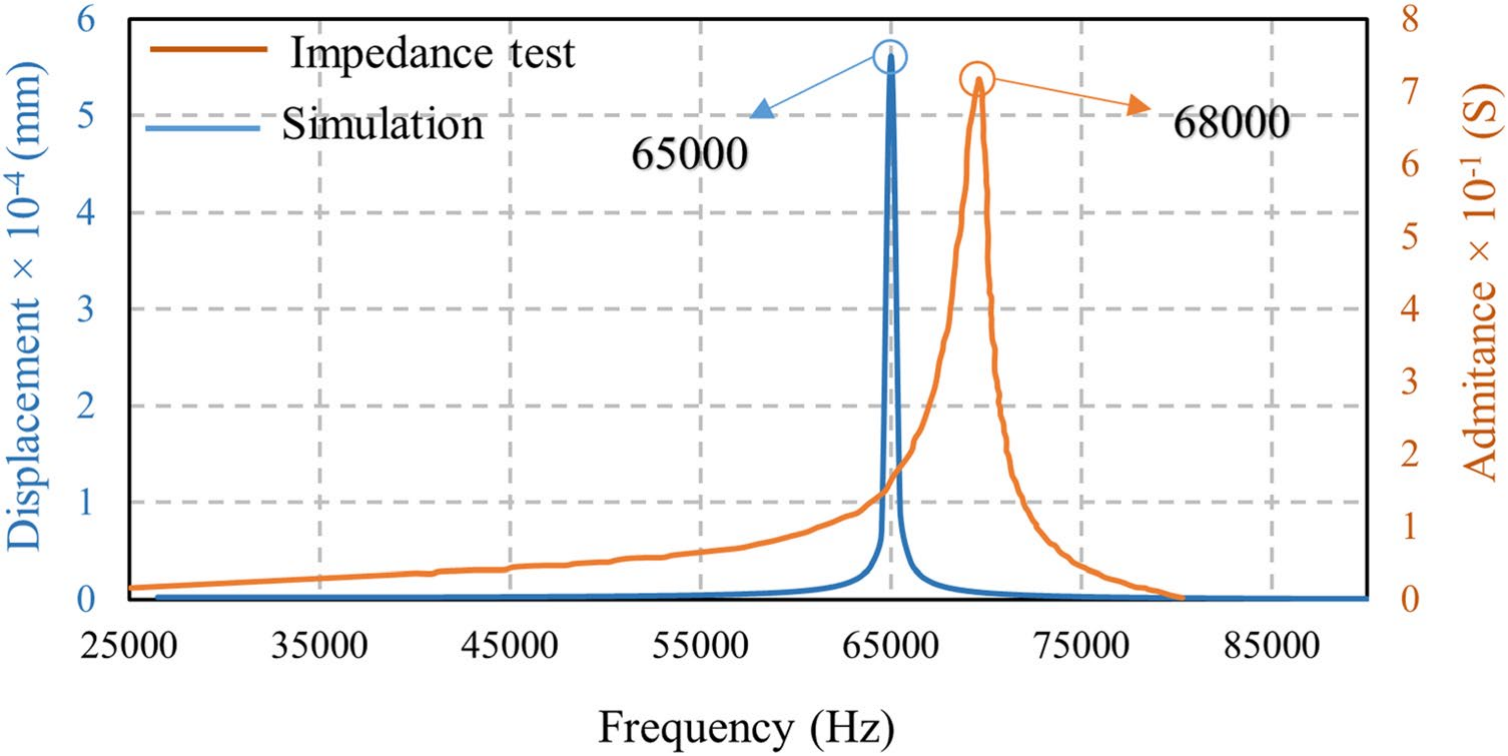
Resonant frequency of piezoelectric stack in the range of 25–85 kHz.

The frequency domain analysis for the free piezoelectric stack with no preload was evaluated in the range of 25–85 kHz and under the impedance analyzer testing. A frequency domain analysis was also conducted for a preloaded piezoelectric stack, as shown in Fig. 12. The impedance analyzer test and simulation reported the resonant frequency of the piezoelectric stack as 68 and 65 kHz, respectively. According to Fig. 12, the resonant frequency value obtained by simulation and experiment were very close (a less than 5% difference) which can be attributed to numerical error.

**Figure 13:**
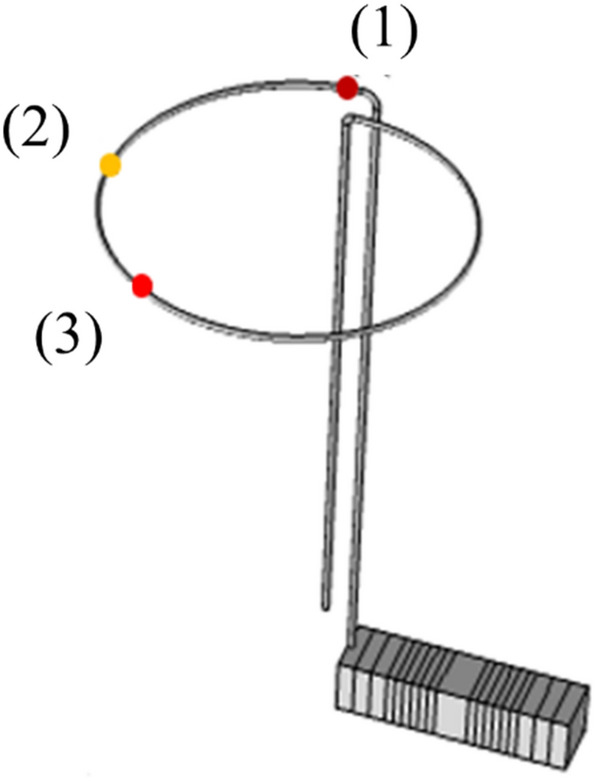
Frequency domain analysis of setup for three different points in wire stator.

Therefore, the stator assembly (piezoelectric stack and wire stator) was modeled in the software, and the model was evaluated in the range of 25–40 kHz. The resonance occurred near 33 kHz. The frequency-domain was assessed at three different points on the wire stator (Fig. 13), which were identical.

For the MDOF actuation of the spherical rotor, stator S_1_ was given a frequency of 31.4 kHz to rotate around axis S_1_, and the stator S_2_ produced no wave. In the next step, the stator S_2_ was given a frequency of 33 kHz to rotate around the axis S_2_, and the stator S_1_ produced no wave. By applying a 3.6-V voltage (7.2 V peak-to-peak) and a 0.2-A output current, they started to vibrate, and the stators S_1_ and S_2_ were simultaneously actuated, leading to the MDOF motion of the motor. Rotational speeds in each state of Fig. 9 are mentioned in Table 3.

**Table 3:** Frequencies of multidirectional rotation.

Axis of rotation	Frequency of S_1_ (kHz)	Frequency of S_2_ (kHz)	Rotational Speed (rpm)
1	31.4	0	44
2	0	33.0	65
1 + 2	31.4	33.0	84

**Figure 14:**
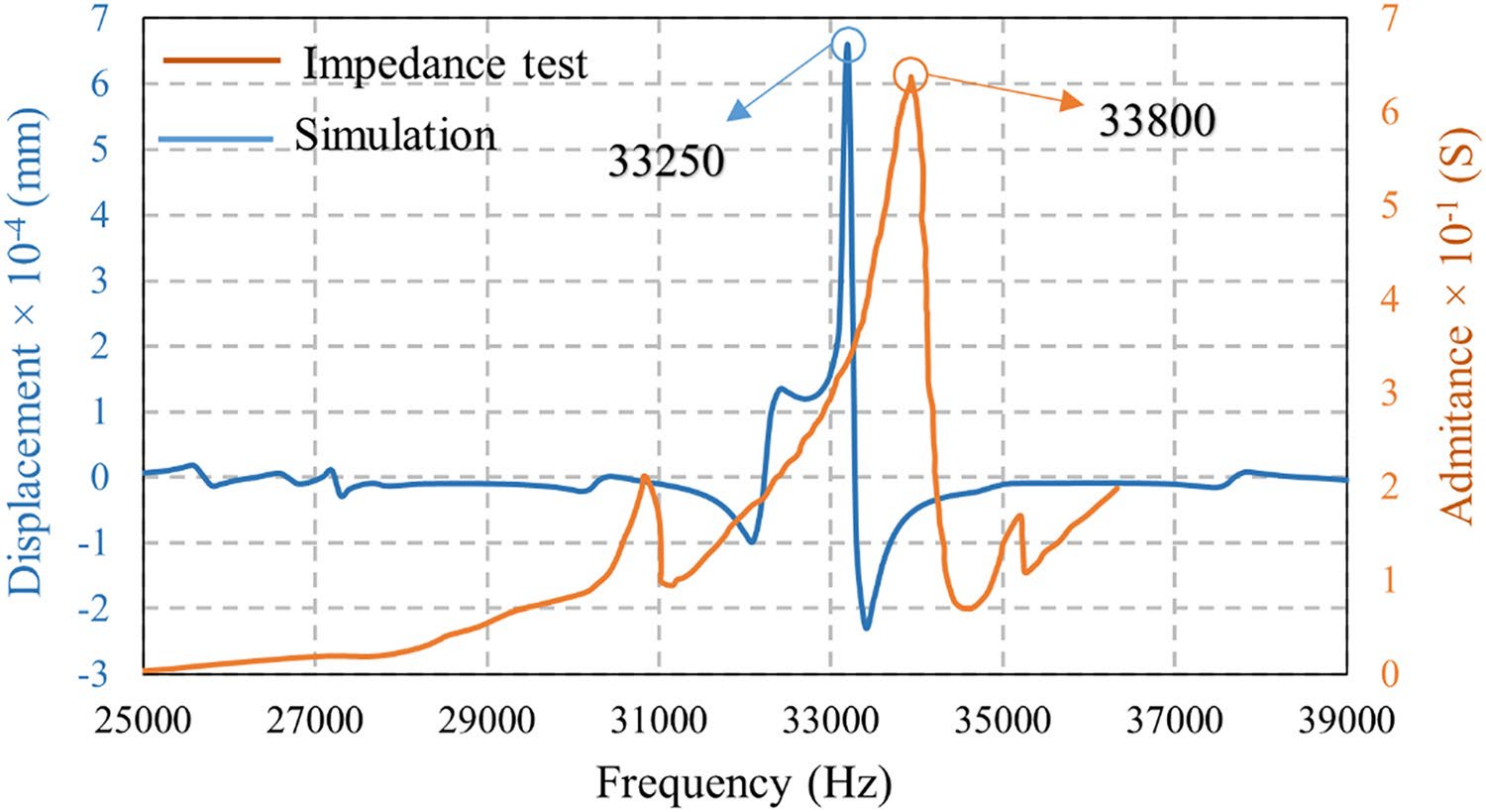
Frequency domain analysis of piezoelectric stack and wire stator as a whole.

Figure 14 compares the stator assembly's experimental and frequency domain simulation curves. The resonant frequency calculated by the impedance analyzer and simulation were 33,800 and 33,250 Hz, respectively. Accordingly, the results were in high agreement (< 3% difference).

**Figure 15:**
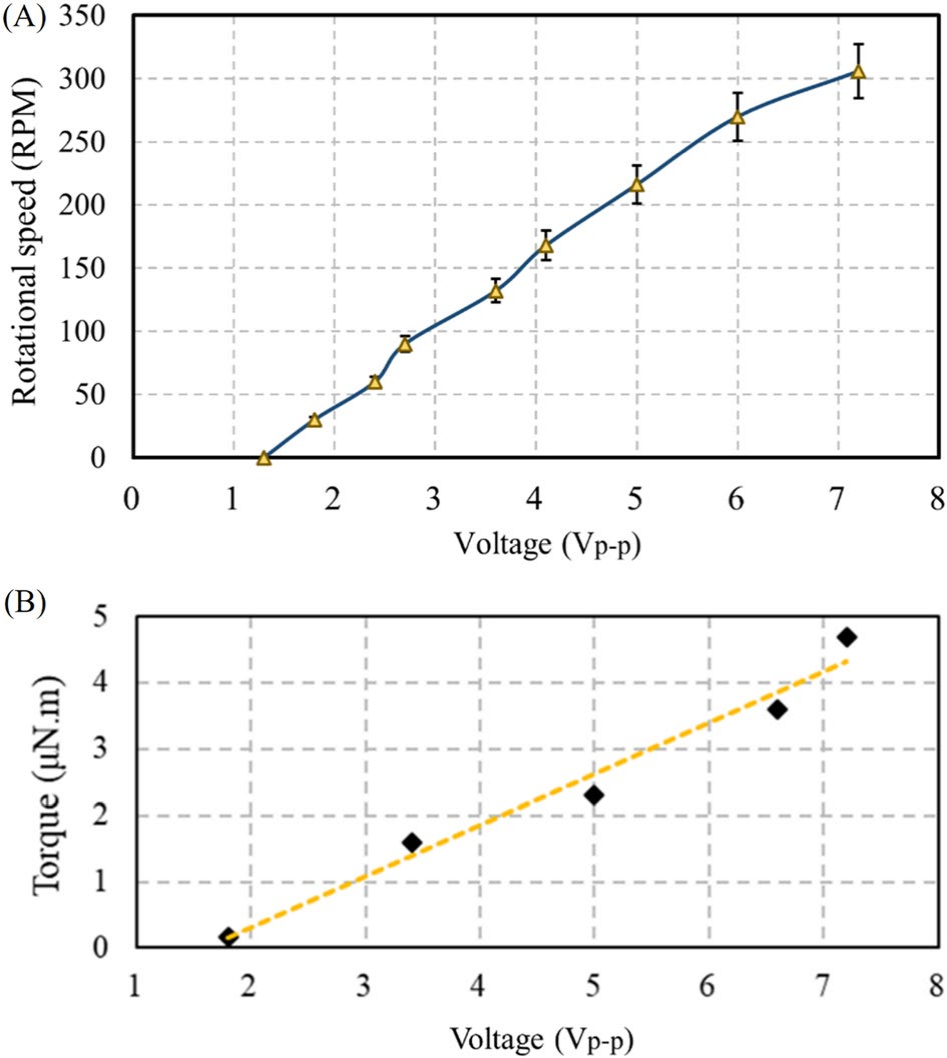
SUSM characteristics: (**A**) rotational speed vs voltage (Peak-to-Peak) (**B**) torque vs voltage (Peak-to-Peak).

As illustrated in Fig. 15A, two stators come in contact with the spherical rotor to drive the motor. As the voltage increased, the vibration amplitude of the stator rose, leading to a higher rotation speed for the rotor. The maximum measured current during the motor operation was 0.2 A, and the maximum power consumption 0.36 W_rms_. The experimental results of output torque versus various exciting voltages were depicted in Fig. 15B, which were measured by the novel measurement method.

**Figure 16:**
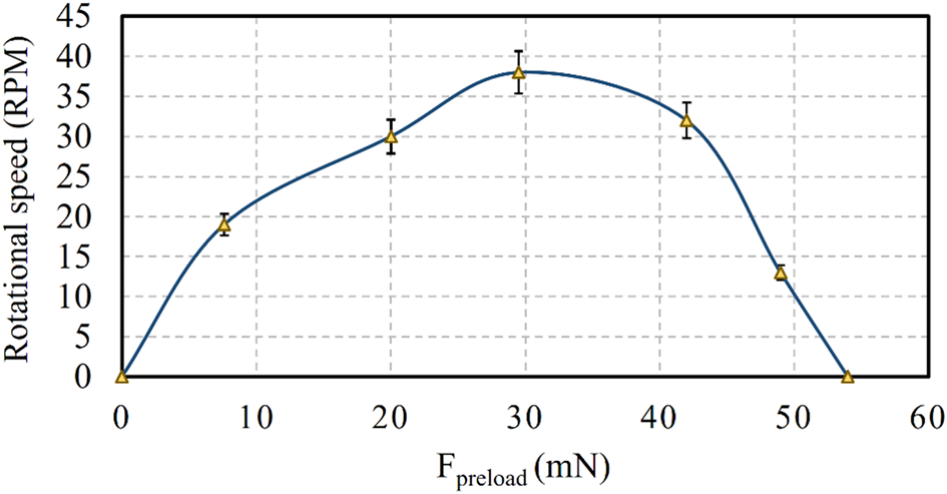
Effect of the preload force on rotational speed.

According to the rheometer measurement, for the rotation speed of 12.5 rpm, the fluid viscosity was 0.26 Pa.s. For different values of parameter *a*, the torque value varied. The results indicated that the torque decreased as the fluid height rose (higher values of *a*). For example, by increasing the fluid (oil) height, the torque decreased from 4.7 μN m to 0.17 μN m. By utilizing this method for torque measurement and knowing the rotational speed, the maximum output power provided by the motor was calculated to be 4.93 μW. Since the friction force affects the preload calculations, Eq. (1) lead to a torque of 0.2 nN.m. Regarding the radius of the spherical rotor (i.e., 0.02 m), the friction force was 40 nN, which is negligible. For the preload force of 29.5 mN, the maximum rotor–stator and rotor rotation speed (i.e., 38 rpm) was achieved. The maximum preload for the spherical rotor was 44.5 mN, for which the rotational speed was 35 rpm. The rotor ceased to move for the preload force of 54 mN (Fig. 16). The characteristics comparison with other multi-axis rotary piezoelectric actuators were listed in Table 4. As previous studies reported voltage in root mean square (rms) form, the voltage of the proposed motor was converted to rms to facilitate the comparison. In summary, the very low driving voltage, simple structure, simple–to–implement and easy-to-control driving circuits for actuation, the capability of using one stator in different vibration modes to move the rotor around different axes under various frequencies, and very high response speed of the system could be mentioned as the advantages of the proposed motor. Optimizing the design of the motor along with the mentioned benefits enables the proposed motor to move a micro camera in microendoscopy and thin-layer sensors in various orientations as specific applications. On the other hand, low stability and resistance to buckling in wire stators due to the low diameter of wires, and very low output torque because of the limitations of the piezoelectric stack to provide sufficient power for large output torque are the drawbacks of the motor. Investigating and optimizing the effective design factors to maximize the output operative parameters could be the subject of future studies.

**Table 4:** The characteristics comparison with other multi-axis rotary piezoelectric actuators.

References	Frequency (kHz)	Voltage (V_rms_)	Speed (rpm)	Torque (mN m)
^25^	58	70	65	3.5
^34^	26.2	35	55	118
^35^	54.3	60	106	17.5
^36^	31.73	120	318	15.3
^26^	40.35	71	29	–
^1^	40.36	400	69.4	2.42
The proposed motor	32.5	2.55	307	0.0047

## Conclusions

This study sought to conceptually design and evaluate a spherical ultrasonic motor numerically and experimentally, in which large transducers were replaced by small piezoelectric stacks as the actuator of the spherical rotor. The new design decreased the motor size and power consumption, and the motor can be excited for any DOF in a single phase. Moreover, different modes of piezoelectric stacks can be utilized for different frequencies, allowing for rotor rotation in various directions. A stator made of single spiral wire was used, improving the motor size and efficiency.

An FE-based modal and frequency domain analysis and the simulation of piezoelectric stacks were carried out. Different vibration modes for piezoelectric stacks and their corresponding frequencies were derived. The resonant frequencies of piezoelectric stacks and the whole motor were 65 and 32.8 kHz. The frequency domain was evaluated at three different points, and their resonant frequency was about 33 kHz. The impedance analyzer test was performed for piezoelectric stacks and the whole assembly, and comparing the results with the simulations showed an error tolerance of less than 5%.

After designing and determining the properties, some experiments were conducted. Regarding the limitations of conventional torque calculation methods, a new method was proposed based on the shear stress caused by the viscous fluid flow over the spherical rotor. The preload force was calculated using the buoyancy force exerted on the immersed rotor. At 32 kHz, the rotational speed of the spherical rotor was directly proportional to voltage. The maximum rotational speed of 306 rpm was achieved with a peak-to-peak voltage of 7.2 V. Maximum torque and preload force were 4.7 μN m and 44.5 mN, respectively. For the preload of 30 mN, the maximum speed was 38 rpm. The results suggest that proposed SUSM has the capability to precise MDOF angular positioning as a topic for future research. Evaluating the effect of design parameters (diameter of the wire, length of the stator arms, number of spirals, etc.) on the overall performance and measurement of the resolution and efficiency of the presented motor could be the subject of future studies.

## Data Availability

All data generated or analysed during this study are included in this published article.
